# Assessing vulnerability of giant pandas to climate change in the Qinling Mountains of China

**DOI:** 10.1002/ece3.2981

**Published:** 2017-04-25

**Authors:** Jia Li, Fang Liu, Yadong Xue, Yu Zhang, Diqiang Li

**Affiliations:** ^1^ Research Institute of Forest Ecology, Environment and ProtectionChinese Academy of Forestry/Key Laboratory of Forest Ecology and Environment of State Forestry AdministrationHaidianBeijingChina

**Keywords:** adaptive conservation strategies, Maxent, nature reserve, suitable habitat, vulnerability

## Abstract

Climate change might pose an additional threat to the already vulnerable giant panda (*Ailuropoda melanoleuca*). Effective conservation efforts require projections of vulnerability of the giant panda in facing climate change and proactive strategies to reduce emerging climate‐related threats. We used the maximum entropy model to assess the vulnerability of giant panda to climate change in the Qinling Mountains of China. The results of modeling included the following findings: (1) the area of suitable habitat for giant pandas was projected to decrease by 281 km^2^ from climate change by the 2050s; (2) the mean elevation of suitable habitat of giant panda was predicted to shift 30 m higher due to climate change over this period; (3) the network of nature reserves protect 61.73% of current suitable habitat for the species, and 59.23% of future suitable habitat; (4) current suitable habitat mainly located in Chenggu, Taibai, and Yangxian counties (with a total area of 987 km^2^) was predicted to be vulnerable. Assessing the vulnerability of giant panda provided adaptive strategies for conservation programs and national park construction. We proposed adaptation strategies to ameliorate the predicted impacts of climate change on giant panda, including establishing and adjusting reserves, establishing habitat corridors, improving adaptive capacity to climate change, and strengthening monitoring of giant panda.

## 1 Introduction

Rapid climate change has been widely recognized as a major threat to biodiversity (Cramer et al., 2014). Compelling evidence has already been presented of the effects of climate change on geographic distributions (Ancillotto, Santini, Ranc, Maiorano, & Russo, 2016; Molina‐Martínez et al., 2016), population dynamics (Auer & Martin, 2013; Lehikoinen et al., 2016), phenological phase (Lučan, Weiser, & Hanák, 2013; Yang & Rudolf, 2010), and evolution (Charmantier & Gienapp, 2014; Koen, Bowman, Murray, & Wilson, 2014), and these impacts are predicted to be exacerbated in future (Rinawati, Stein, & Lindner, 2013; Urban, 2015). Projected change rates of climate are now getting faster than they were in the past (IPCC, 2014). If global warming is not effectively controlled, a mean increase in global temperature of >2.0°C could be the result (2.0°C is defined as “dangerous”; UNFCCC, 2015), and 15%–35% of global species could be committed to extinction (Thomas et al., 2004). Although the impact of climate change on the extent and rate of species extinction is still controversial, it is clear that the trend of global warming will accelerate the extinction risk for species (Malcolm, Liu, Neilson, Hansen, & Hannah, 2006; Pereira et al., 2010; Urban, 2015).

Faced with an irrefutable crisis of biodiversity loss, it is imperative to assess the vulnerability of species to future climate change, and adopt conservation strategies to mitigate the harmful impacts of climate change on these species (Heikkinen, Luoto, Leikola, Pöyry, & Settele, 2010; Polaina, Revilla, & González‐Suárez, 2016; Tuberville, Andrews, Sperry, & Grosse, 2015; Williams, Shoo, Isaac, Hoffmann, & Langham, 2008). Assessments of species vulnerability to climate change are usually based on available information of the species being assessed (Glick, Stein, & Edelson, 2011; Pacifici et al., 2015; Rowland, Davison, & Graumlich, 2011). A few tools and approaches have been developed to assess species’ vulnerability to climate change, such as vulnerability indices (Bagne, Friggens, & Finch, 2011; Foden et al., 2013; Young et al., 2015), mechanistic distribution models (Kearney & Porter, 2009; Monahan, 2009), and bioclimatic envelope models (Lawler, Shafer, & Bancroft, 2010; Pearson et al., 2014). Bioclimatic envelope models are one of the most common approaches, because they generally require only robust data on species ranges and an associated climate database (Rowland et al., 2011). Spatial shifts in climatically suitable habitat under climate change scenarios are then forecasted (Kane, Burkett, Kloper, & Sewall, 2013; Rowland et al., 2011; Thuiller, Lavorel, & Araújo, 2005). Identifying species’ potential range shifts is crucial for management and conservation of vulnerable species in a changing climate (Heikkinen et al., 2010).

The giant panda (*Ailuropoda melanoleuca*) is probably one of the world's most treasured endangered species (Wei et al., 2015). Its habitat is currently restricted to six isolated mountain ranges in Sichuan, Shaanxi, and Gansu provinces in south‐central China (State Forestry Administration, 2015). The giant panda was listed as an endangered species by the International Union for Conservation of Nature (IUCN) in 1996 (IUCN, 1996) due to their limited geographic range, the risk of small and isolated populations, low reproductive rates, habitat loss, and diet specialization (Swaisgood, Wang, & Wei, 2016; Wang, Ye, Skidmore, & Toxopeus, 2010; Wei et al., 2015). A narrow geographic distribution makes them highly susceptible to climate change (Liu, Guang, Dai, Li, & Gong, 2016; Songer, Delion, Biggs, & Huang, 2012). Over the past decades, the Chinese government implemented many conservation programs to protect giant panda, such as establishment of reserves (State Forestry Administration, 2015), the panda monitoring project (Wei et al., 2015), and the Grain‐to‐Green program (Li et al., 2013). From 1988 to 2015, the population of giant panda grew from 1,114 to 1,864 (State Forestry Administration, 2015), and the species has been downlisted from “Endangered” to “Vulnerable” in the IUCN Red List of Threatened Species (Swaisgood et al., 2016). The Chinese government announced that giant panda conservation programs will continue and will establish national parks in the giant panda's range to specifically strengthen further conservation of giant panda (State Forestry Administration, 2016a). Therefore, a major motivation for assessing the vulnerability of giant panda is to provide adaptive strategies for conservation programs and development of national parks to reduce effectively potential climate‐related threats to the species.

In this study, we use the maximum entropy model (i.e., Maxent, Phillips, Anderson, & Schapire, 2006) to predict the habitat suitability, to assess vulnerability of the giant panda to climate change, and to identify the potential refuges and corridors. Furthermore, we propose the conservation strategies for the species and provide fundamental information for establishing giant panda national parks in the Qinling Mountains of China.

## 2 Methods

### 2.1 Study area

The study area is located in the Qinling Mountains (106°30′–108°05′E, 32°40′–34°35′N) in Shaanxi Province in China. The Qinling Mountains are characterized by a specific geographic system in terms of topography and climate, and include the boundary between the temperate and subtropical zones (Zhao, Zhang, & Dong, 2014). The mountains rise from 222 m to 3,734 m, with a gentler gradient on the southern slope; however, their northern face is generally steep (Pan et al., 2001). Regarding the differences in climate between northern and southern China, the southern slope is generally warmer and moister than the northern face, and climatic conditions vary with elevation gradient (Pan et al., 2001). Deciduous broadleaf and subtropical evergreen forests mainly inhabit at low elevation; temperate broadleaf and subalpine coniferous forests inhabit at mid‐elevation; and subalpine scrub meadows inhabit at high elevation (State Forestry Administration, 2006). A population of 345 giant pandas was estimated to inhabit the Qinling Mountains (State Forestry Administration, 2015). A total of 19 nature reserves (including eight national and nine provincial nature reserves, and two at the application stage) have been established to protect giant panda and their habitat in this region (State Forestry Administration, 2015).

### 2.2 Data preparation

**Figure 1:**
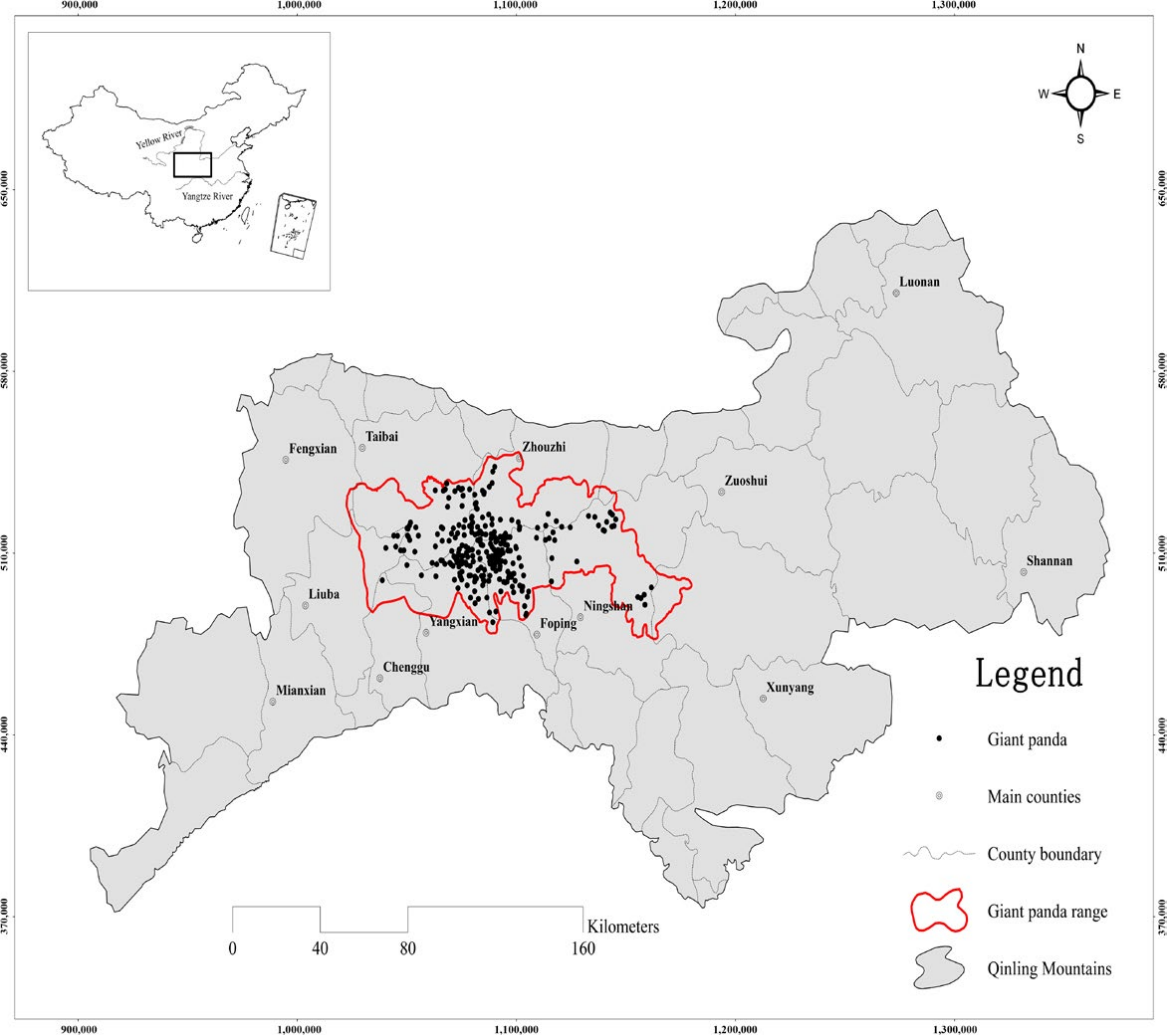
Distribution of giant pandas in Qinling Mountains

Locations of giant panda's signs (including feces, dens, bed sites, and footprints, *N *=* *273) were obtained from the 3rd National Survey Report on Giant Panda in China (State Forestry Administration, 2006; Figure 1). Model parameters require an unbiased sample; therefore, we filtered the presence points by randomly choosing one record per 1 × 1 km cell. To correct the effect of sample selection bias on predictive performance (Phillips et al., 2009), we created 10,000 random points as target‐group background points in our study area and the random points were generated from any forest in the Qinling Mountains (the forest data were derived from global land cover, which were interpreted by the United Nations Food and Agricultural Organization; Charmantier & Gienapp, 2014; Tateshi et al., 2014).

Nineteen bioclimatic variables at 30 s resolution (~1 km) were obtained from the WorldClim database (Hijmans, Cameron, Parra, Jones, & Jarvis, 2005) for current climatic (average for 1950–2000) and future climatic scenarios for the 2050s (average for 2041–2060; available at http://www.worldclim.org/version1). The future climate data applied in this study comprised of IPCC‐CMIP5 climate projection from the Met Office Hadley Center for climate change coupled model (HadGEM_2_‐AO) under the representative concentration pathway (RCP) 4.5 (Baek et al., 2013). For the 2050s, the average increase in global temperature of 0.9–2.0°C under RCP4.5 would fall within a 2°C global warming limit (UNFCCC, 2015). The time horizon of the 2050s was selected for being a date far enough in future for significant changes to have occurred (Young et al., 2012).

Other environmental variables were also used to construct the panda distribution models (Fan et al., 2011; Loucks et al., 2003). The densities of rivers, roads, and settlements were obtained from a 1:1,000,000 map of China (National Geomatics Center of China, data are available at http://atgcc.sbsm.gov.cn). Elevation data were derived from a digital elevation model with a resolution of 30 s, obtained from the WorldClim database (Appendix 1). Because nonclimate variables (i.e., densities of roads, rivers, and settlements) were not available for the 2050s, we used the same variables in projections for the 2050s.

All spatial layers were resampled into resolution of 1 × 1 km and projected to an equal area projection (Asia North Albers Equal Area Conic) using ArcGIS 10.1 (ESRI Inc., Redlands, CA, USA). We then calculated correlation coefficients between variables and eliminated one variable from each pair that was strongly correlated (|*r*| > .8; Cord, Klein, Mora, & Dech, 2014; Lemke, Hulme, Brown, & Tadesse, 2011). Thirteen variables (annual precipitation, annual temperature range, density of rivers, density of roads, density of settlements, elevation, mean diurnal range, min. temperature of coldest month, precipitation of warmest quarter, precipitation seasonality, precipitation of driest quarter, precipitation of driest month, and temperature constancy) which were the most biologically meaningful for giant pandas were retained (Appendix 2; Li, Xu, Wong, Qiu, & Li, 2015; Songer et al., 2012). Subsequently, we first input thirteen environmental variables layer into the Maxent model. Then, we input the set of most important variables based on permutation importance obtained from first model output, to construct the giant panda finally distribution model, and rerun the Maxent models.

### 2.3 Habitat suitability model

We used the Maxent software (version 3.3.3k) to build the habitat suitability model for the giant panda. This approach is considered one of the best performing algorithms in predicting species distribution with presence‐only data (Elith, Phillips, Hastie, Dudík, & Chee, 2011). It has been extensively applied to project species range shifts under climate change (Li, Clinton, et al., 2015; Lei, Xu, Cui, Guang, & Ding, 2014; Songer et al., 2012). Maxent estimates species distributions by finding the probability distribution of maximum entropy, subject to the constraints of the data that are available (Phillips et al., 2006). Maxent also estimates the importance of variables and contributions representing the degree to which each variable has contributed to the model, based on jackknife tests. We divided the occurrence data of giant panda into training sets (75%) for model building, and testing sets (25%) for model evaluation, and conducted a subsample procedure (Khatchikian, Sangermano, Kendell, & Livdahl, 2011; Wisz et al., 2008) to evaluate the habitat suitability model by performing 15 replications in Maxent.

Model performance was measured using the area under the receiver operating characteristic curve (AUC). An AUC value closer to 1 represents near perfect performance of the model (Phillips et al., 2006). The output of Maxent comprised continuous values between 0 and 1 that were considered as probabilities of species’ occurrence. We then convert these values to presence and absence predictions, based on the threshold values that maximized training sensitivity plus specificity (Liu, Berry, Dawson, & Pearson, 2005; Songer et al., 2012). The cells with probability values above the threshold value were selected as suitable habitat for the giant panda. We then removed patches <4 km^2^ and >0.5 km distance from the nearest patch based on the minimum home range size and the average daily dispersal ability of specie (Pan et al., 2001). A Mann–Whitney *U* test was used to examine the difference in mean elevation of suitable habitat between current and the 2050s. Statistical analyses were conducted using the SPSS 19.0 software (IBM Inc., USA).

### 2.4 Gap analysis of nature reserves

The Gap analysis of protection of biodiversity is a powerful and efficient step to first assess the protection of biodiversity on a coarse‐filter scale (Scott et al., 1993). The current and projected suitable habitat were overlapped with the boundaries of established nature reserve networks, to explore the conservation effectiveness of these reserves in protecting giant pandas under climate change (Feeley & Silman, 2016).

### 2.5 Vulnerability assessment

The identification of vulnerable habitat of species under climate change scenario is important for decision‐making in adaptive conservation management (Guisan, Tingley, Baumgartner, Naujokaitis‐Lewis, & Sucliffe, 2013). Suitable habitat changes between the current and the 2050s illustrate the locations that likely would be vulnerable, categorized as follows:

1. Unchanged suitable habitat: the area where suitable habitat overlapped between current and the 2050s;
2. Vulnerable habitat: the area where current suitable habitat transferred to unsuitable habitat by the 2050s;
3. New increased suitability habitat: the area where current unsuitable habitat changed to suitable habitat by the 2050s;
4. Unsuitable habitat: the area where unsuitable habitat overlapped between current and the 2050s.

We used three indicators to assess the impacts of climate change on giant panda: (1) percentage of area change (AC); (2) percentage of loss area of current suitable habitat (SH_c_); and (3) percentage of increased area of the 2050s’ suitable habitat (SH_f_). Indicators calculated as follows:

(1) $$\text{AC}\;=\;\frac{A_{f}\;-\;A_{c}}{A_{c}}\;\times \;100\%,$$

(2) $${\text{SH}}_{c}\;=\;\frac{A_{c}\;-\;A_{\mathrm{fc}}}{A_{c}}\;\times \;100\%,$$

(3) $${\text{SH}}_{f}\;=\;\frac{A_{f}\;-\;A_{\mathrm{fc}}}{A_{f}}\;\times \;100\%.$$

In these formulas, *A*_f_ is area of projected suitable habitat for pandas under the 2050s’ climatic scenario; *A*_c_ is the area of projected current suitable habitat; *A*_fc_ is the overlapped distribution space between current and the 2050s (Duan, Kong, Huang, Vaerla, & Ji, 2016; Levinsky, Skov, Svenning, & Rahbek, 2007; Thuiller, Lavorel, Araújo, Sykes, & Prentice, 2005).

## 3 Results

### 3.1 Species distribution model

**Figure 2:**
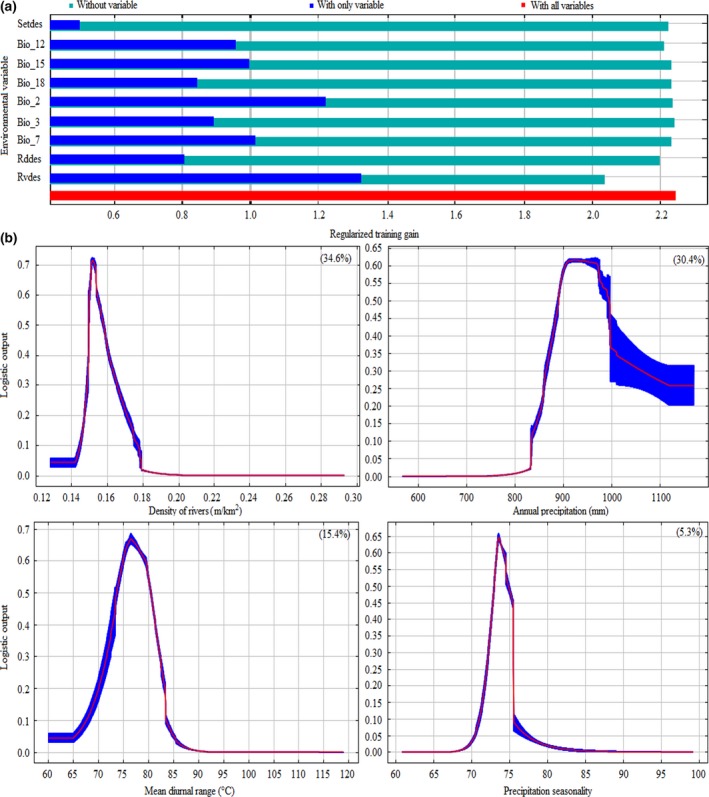
Results of Maxent models: (a) Jackknife test of variable importance. Codes of the variables are found in Appendix 1; and (b) marginal response curves of environmental variables in Maxent model of giant panda

In the Maxent model, 256 presence points and nine variables were finally used as model parameters to construct the giant panda distribution model. The average training AUC was 0.967 ± 0.001, and the average testing AUC was 0.961 ± 0.005. The permutation importance of variables in the model as ranked from the highest to the lowest were as follows: density of rivers (34.6%), annual precipitation (30.4%), mean diurnal range (15.4%), precipitation seasonality (5.3%), precipitation of warmest quarter (4.4%), density of roads (3.6%), annual temperature range (3.1%), density of settlements (2.0%), and temperature constancy (1.3%; Figure 2). The average threshold for the probability of presence at maximum training sensitivity plus specificity was .1434. We then defined the cells with probability values greater than .1434 as suitable habitat for giant panda.

### 3.2 Suitable habitat change

**Figure 3:**
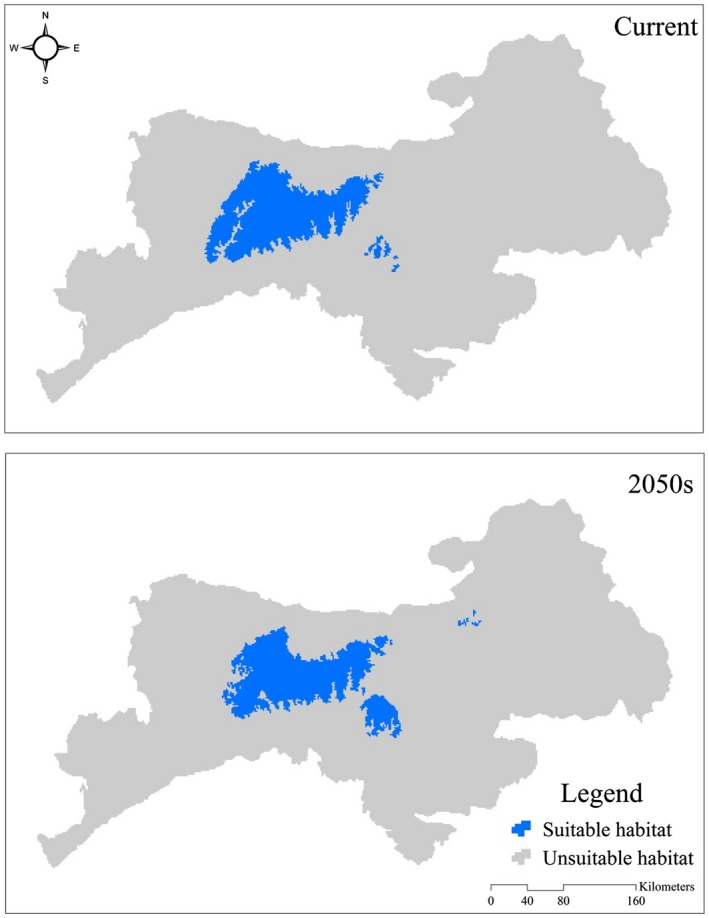
Predicted current and the 2050s’ suitable habitat for giant panda in Qinling Mountains

Under the current conditions, area of suitable habitat for giant panda in the Qinling Mountains was 4,810 km^2^. Current suitable habitat for giant panda is distributed in Chenggu, Foping, Liuba, Ningshan, Taibai, Yangxian, and Zhouzhi counties. For the 2050s, a reduction to 4,529 km^2^ (AC = −5.8%) in the area of suitable habitat was projected, and mainly distributed among Foping, Ningshan, Taibai, Yangxian, and Zhouzhi counties (Figure 3). Climate change would result in the shift of suitable habitat for giant panda to higher elevations. The mean elevation of suitable habitat in the 2050s was projected to be 1,870.57 ± 418.57 m, which was significantly higher (*Z* = −3.877, *p *=* *.000) than that of current suitability habitat (1,837.41 ± 432.24 m).

### 3.3 Gap analysis of nature reserve network

**Figure 4:**
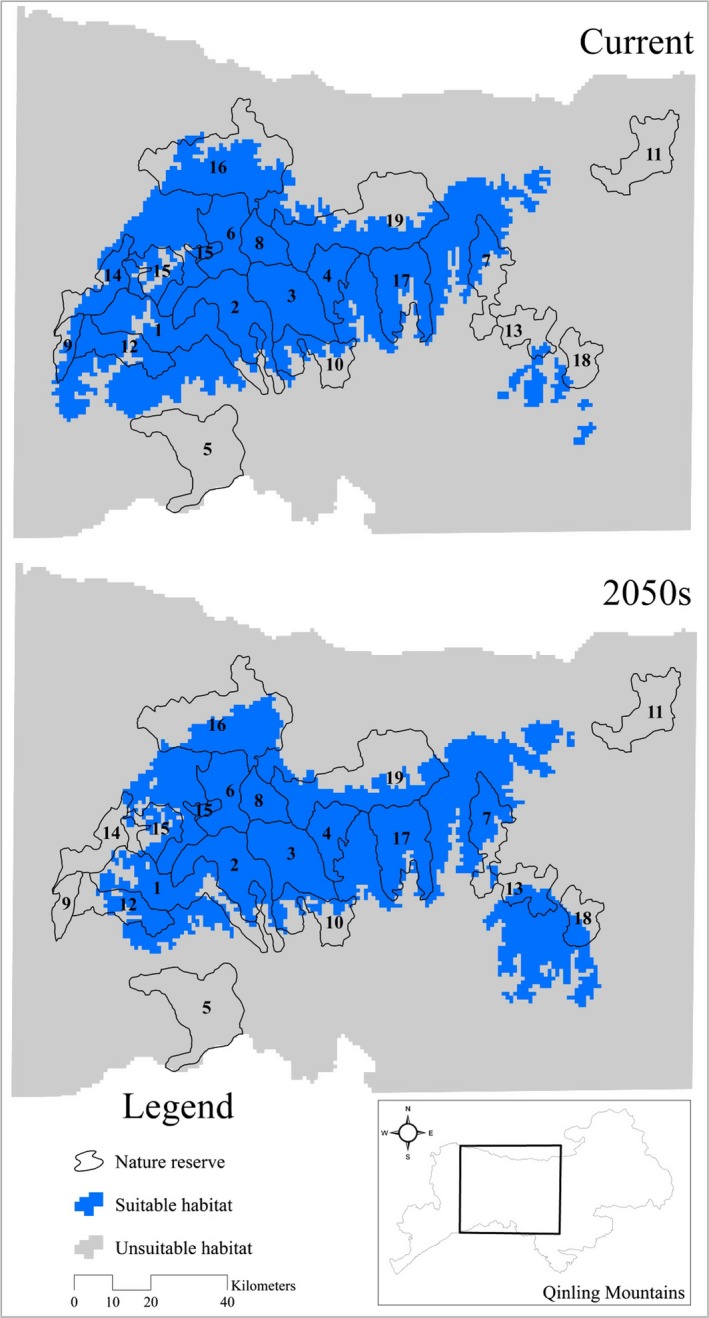
Gap analysis of the giant panda in Qinling Mountains. Codes of the reserves: 1—Banqiao, 2—Changqing, 3—Foping, 4—Guanyinshan, 5—Hangzhongzhuhuan, 6—Huangbaiyuan, 7—Huangguanshan, 8—Laoxiancheng, 9—Motianling, 10—Niangniangshan, 11—Niubeiliang, 12—Panlong, 13—Pingheliang, 14—Sanyuan, 15—Taibainiuweihe, 16—Taibaishan, 17—Tianhuashan, 18—Yingzuishi, 19—Zhouzhi

Nature reserves protect 61.73% of current suitable habitat and 59.23% of suitable habitat in the 2050s (Table 1, Figure 4). In the 2050s, giant panda suitable habitat is predicted to suffer loss in nine nature reserves, among which Banqiao (AC = −21.66%), Motianling (AC = −100%), Niangniangshan (AC = −22.20%), Panlong (AC = −35.44%), Sangyuan (AC = −92.46%), Tiabaishan (AC = −22.83%), and Zhouzhi (AC = −9.87%) nature reserves estimated to suffer the greatest loss of suitable habitat in the 2050s. Climate change will increase the extent of the panda's distribution, mainly in the Huangguanshan, Pingheliang, and Yingzuishi nature reserves (Table 1).

**Table 1:** Projected change in suitable habitat of giant pandas in nature reserves

Nature reserve	Suitable habitat area/(km^2^)		Percentage of area change (AC)
	Current	2050s	
1—Banqiao	309.20	242.23	−21.66
2—Changqing	307.21	295.96	−3.66
3—Foping	300.00	300.00	0.00
4—Guanyinshan	148.44	148.44	0.00
5—Hanzhongzhuhuan	0.00	0.00	—
6—Huangbaiyuan	210.79	210.79	0.00
7—Huangguanshan	81.28	129.75	59.64
8—Laoxiancheng	120.57	120.57	0.00
9—Motianling	47.56	0.00	−100.00
10—Niangniangshan	96.49	75.24	−22.02
11—Niubeiliang	0.00	0.00	—
12—Panlong	158.13	102.10	−35.44
13—Pingheliang	12.36	58.54	373.63
14—Sangyuan	120.05	9.05	−92.46
15—Taibainiuweihe	106.98	102.77	−3.94
16—Taibaishan	348.93	269.28	−22.83
17—Tianhuashan	273.31	285.90	4.60
18—Yingzuishi	5.78	41.33	615.41
19—Zhouzhi	322.31	290.50	−9.87

### 3.4 Vulnerability assessment

**Figure 5:**
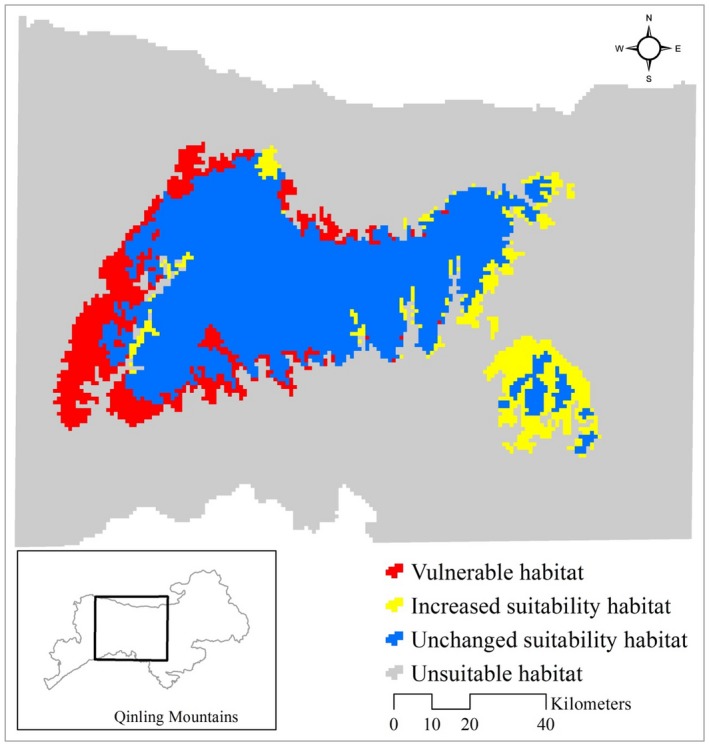
Vulnerability analysis of giant panda suitable habitat in Qinling Mountains

Our predicted 3,823 km^2^ of unchanged suitable habitat is mainly distributed in Foping, Ningshan, Taibai, Yangxian, and Zhouzhi counties. We predicted 987 km^2^ (SH_c_ = 20.52%) of current suitable habitat distributed in Chenggu, Taibai, and Yangxian counties is expected to become vulnerable habitat. Interestingly, our results also revealed that there was an increase in the extent of suitable habitat (706 km^2^, SH_f_ = 15.89%) in Ningshan country (Figure 5).

## 4 Discussion

Over the past several decades, giant pandas have been exposed to several threats to their survival, such as bamboo flowering, extensive poaching, and habitat destruction (Li, Guo, Yang, Wang, & Niemelä, 2003; Pan et al., 2001). However, the Chinese government has conducted giant panda conservation programs, and many of the key threats have been mitigated (Wei et al., 2015; Zhu et al., 2013). At present, human disturbances (e.g., roads, construction projects, ecotourism, and environmental pollutants) and climate change are considered as the paramount threats that degrade and fragment panda habitat (Wei et al., 2015). Particularly, the impacts of climate change on giant panda may have negative impacts on current conservation efforts (Shen, Pimm, Feng, Ren, & Liu, 2015). Therefore, assessing vulnerability is the key step to develop proactive strategies to reduce the impacts of climate change on the giant panda.

Based on the model output, under a mild climate change scenario (RCP 4.5), 20.52% (SH_c_) of current suitable habitat of giant panda is projected to transfer to unsuitable habitat, particularly in the southwestern region of the Qinling Mountains (i.e., Chenggu and Liuba counties). Climate change associated with suitable habitat fragmentation would present another conservation challenge for this species (Holyoak & Heath, 2016; Li, Clinton, et al., 2015). Current habitat connectivity in southwestern portion of Qinling Mountains is relatively low, and these areas are predicted to experience greatest loss by the 2050s due to climate change, thereby emphasizing the need for a regional conservation strategy for giant panda conservation to protect these areas, and constructing migration corridors to facilitate the dispersal of southwestern populations to large core areas. Fortunately, Ningshan county is predicted to have considerable areas of newly suitable habitat for giant panda. However, migration into the new areas may be impeded by both natural and artificial barriers (e.g., rivers, roads, and human settlements; Fan et al., 2011; Sun et al., 2007). Therefore, proactive measures for habitat restoration should be taken to protect and improve the habitat for the species, and construct migration corridors to facilitate the dispersal of a greater number of giant panda to these new suitable habitats (that currently have a relatively small population of giant panda; Sun et al., 2007).

An assessment of the impact of climate change on species is a critical initial step in implementing the adaptation planning process (Rowland et al., 2011). Some nature reserves, among which planning had been done decades in advance, need to be re‐evaluated considering climate change (Bellard, Bertelsmeier, Leadley, Thuiller, & Courchamp, 2012; Hansen, Hoffman, Drews, & Mielbrecht, 2010). Our results revealed that the loss of giant panda suitable habitat would affect the conservation effectiveness of the existing giant panda reserves. These reserves do not adequately protect the current suitable habitat of giant panda, nor will they protect future potential suitable habitat. Coping strategies to deal with potential threats, particularly in those nature reserves (i.e., Banqiao, Motianling, Panlong, Sanyuan) that would suffer the greatest loss of suitable habitat under future climate change, require further in‐depth study. Meanwhile, three provincial nature reserves located in Ningshan county are also urgent need to improve their conservation effectiveness against climate change, because they currently support a small population of giant panda (Sun et al., 2007), but are isolated from the network of large reserves, and have low levels of protection (Figure 4).

Vulnerability assessments can provide information about the locations that are vulnerable to climate change (Levinsky et al., 2007) and broadscale guidance to direct conservation efforts (Dubois, Caldas, Boshoven, & Delach, 2011; Rowland et al., 2011). Based on our vulnerability assessment, protection needs to prioritize habitat in which the maximum effects of climate change are predicted to occur, namely the vulnerable areas. These regions are predicted to suffer from large range contractions under climate change and present the greatest risk to the persistence of giant panda in the 2050s. Furthermore, vulnerability assessments are able to identify the potential climatic refuges for giant panda within Qinling Mountains range, namely unchanged and new increased suitability habitat (Ashcroft, 2010; Li et al., 2016), and these areas may facilitate species persistence during periods of climatic stress.

### 4.1 Conservation implications

As a flagship species in China, the government of China has listed the giant panda in the key program of biodiversity conservation (Ministry of Environmental Protection et al., 2011), and will conduct giant panda conservation programs and establish national parks specifically for protecting the species in Shaanxi, Sichuan, and Gansu provinces (State Forestry Administration, 2016b). Thus, assessment of vulnerability provided key information in designing effective adaptation strategies to cope with the impacts of future climate change for national parks development. Our results suggest the following adaptation strategies to ameliorate the predicted impacts of climate change on giant panda in Qinling Mountains:

#### 4.1.1 Establishing new reserves

**Figure 6:**
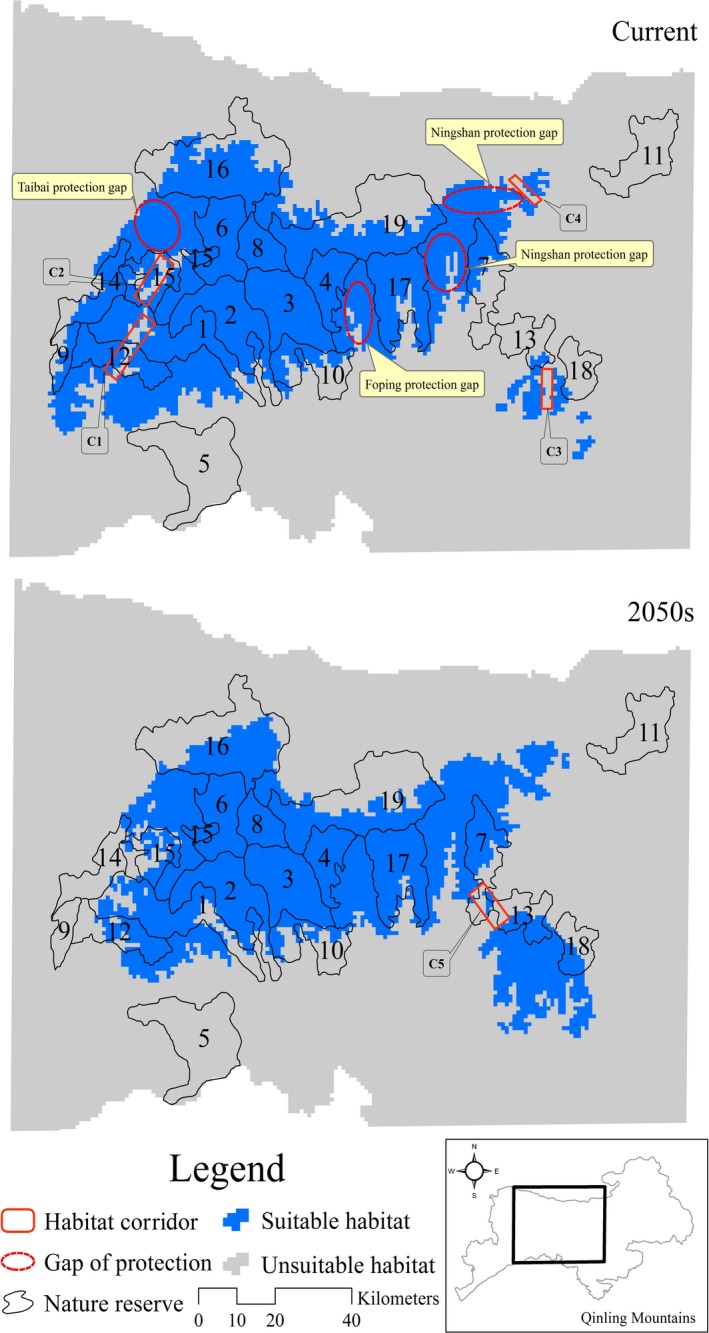
Protection gaps and habitat corridors giant pandas in Qinling Mountains. C1–C5 indicates habitat corridors

Gap analysis showed the distribution of current suitable habitat in Foping, Ningshan, and Taibai counties is largely unprotected, leaving significant gaps in the conservation network, and suitable habitat distributed in these areas will be discrete and fragmentated by the 2050s (Figure 6). Therefore, new reserves need to be established in these regions to improve the connectivity of habitat.

#### 4.1.2 Adjusting reserves

An adjustment of range to the existing nature reserves also might be necessary, where habitat shift is observed within the reserves and in their vicinities. For example, it might be necessary to enlarge the protected area of Huangguanshan to connect with Tianhuashan nature reserve. Similarly, increase in area in Pingheliang and Yingzuishi nature reserves may be needed, to protect their surrounding suitable habitat of giant panda.

#### 4.1.3 Establishing habitat corridors

Establishing migration corridors in juncture of Chenggu, Taibai and Yangxian counties (C1 and C2), and Ningshan county (C5; Figure 6) to increase chances for the small population of these areas to larger suitable areas, and enable giant panda to escapes from unsuitable climatic conditions. We also need to establish habitat corridors in Ningshan county (C3 and C4) to enhance habitat connectivity in these areas.

#### 4.1.4 Improving adaptive capacity to climate change

Reducing nonclimate stressors (such as invasive species, human activities, pollution, disease, and other stressors) will improve the impact on the ability of specie to adapt to climate change (Gross, Watson, Woodley, Welling, & Harmon, 2015). Such as invasive species, anticipatory actions might focus on identifying invasive species likely to expand their ranges in response to climate change, and establishing early‐detection and rapid response protocols designed to keep them from invading sensitive areas.

#### 4.1.5 Strengthening monitoring on giant panda

Many nature reserves just started to consider strategies to adapt to climate change when they made their master plans. We do not fully understand how giant panda will respond to those strategies and what management measures might be effective. Therefore, a standardized monitoring program is necessary for nature reserves to collect information of climate change impacts on panda and monitor the responses of the species to the strategies.

## Conflict of Interest

None declared.

## Appendix 1 The code and description of environmental variables, the min, max, mean, and *SD* values are calculated over the entire Qinling Mountains geographic extent

### 1.1

**Table:**

Variable	Description	Unit	Type	Min	Max	Mean	*SD*
Bio1	Annual mean temperature	°C	Continuous	−1.3	16.9	11.1	2.5
Bio2	Mean diurnal range	°C	Continuous	6.5	11.4	8.9	0.8
Bio3	Temperature constancy		Continuous	21.0	31.0	26.8	1.4
Bio4	Temperature seasonality		Continuous	6,904.0	9,944.0	8,272.1	413.7
Bio5	Max temperature of warmest month	°C	Continuous	12.3	33.7	27.2	2.9
Bio6	Min temperature of coldest month	°C	Continuous	−15.6	1.1	−5.4	2.2
Bio7	Annual temperature range	°C	Continuous	27.9	39.1	32.6	1.7
Bio8	Mean temperature of wettest quarter	°C	Continuous	6.9	26.3	20.4	2.7
Bio9	Mean temperature of driest quarter	°C	Continuous	−10.6	6.5	0.0	2.3
Bio10	Mean temperature of warmest quarter	°C	Continuous	7.8	27.5	21.6	2.7
Bio11	Mean temperature of coldest quarter	°C	Continuous	−10.6	6.5	0.0	2.3
Bio12	Annual precipitation	mm	Continuous	549.0	1,119.0	818.4	73.2
Bio13	Precipitation of wettest month	mm	Continuous	97.0	197.0	149.4	13.0
Bio14	Precipitation of driest month	mm	Continuous	4.0	13.0	7.4	1.8
Bio15	Precipitation seasonality		Continuous	64.0	96.0	72.1	4.6
Bio16	Precipitation of wettest quarter	mm	Continuous	280.0	559.0	417.0	41.1
Bio17	Precipitation of driest quarter	mm	Continuous	14.0	46.0	27.6	6.1
Bio18	Precipitation of warmest quarter	mm	Continuous	247.0	492.0	369.3	33.0
Bio19	Precipitation of coldest quarter	mm	Continuous	14.0	46.0	27.6	6.1
Alt	Elevation above sea level	m	Continuous	189.0	3,637.0	1,225.1	479.8
Rddes	Density of roads	km/km^2^	Continuous	0.1	0.7	0.3	0.1
Rvdes	Density of rivers	m/km^2^	Continuous	0.1	0.3	0.2	0.0
Setdes	Density of settlements	#/km^2^	Continuous	0.3	1.4	0.7	0.1

## Appendix 2 Analysis of correlation coefficient of the environmental variables

### 2.1

**Table:**

Variables	Bio1	Bio2	Bio3	Bio4	Bio5	Bio6	Bio7	Bio8	Bio9	Bio10	Bio11	Bio12	Bio13	Bio14	Bio15	Bio16	Bio17	Bio18	Bio19	Alt	Rddes	Rvdes	Setdes
Bio1	1.000																						
Bio2	.717	1.000																					
Bio3	.719	.839	1.000																				
Bio4	.365	.660	.182	1.000																			
Bio5	.981	.808	.723	.524	1.000																		
Bio6	.912	.416	.601	−.022	.823	1.000																	
Bio7	.513	.863	.471	.943	.663	.120	1.000																
Bio8	.989	.770	.699	.494	.996	.848	.622	1.000															
Bio9	.975	.599	.714	.150	.917	.976	.316	.933	1.000														
Bio10	.982	.784	.689	.534	.997	.824	.656	.999	.916	1.000													
Bio11	.975	.599	.714	.150	.917	.976	.316	.933	1.000	.916	1.000												
Bio12	−.447	−.652	−.380	−.663	−.558	−.172	−.748	−.499	−.300	−.529	−.300	1.000											
Bio13	−.413	−.565	−.298	−.618	−.523	−.177	−.680	−.471	−.283	−.495	−.283	.875	1.000										
Bio14	−.339	.114	−.110	.338	−.261	−.559	.281	−.251	−.437	−.234	−.437	.168	.179	1.000									
Bio15	.016	−.352	−.023	−.626	−.094	.298	−.556	−.103	.154	−.123	.154	.021	.182	−.803	1.000								
Bio16	−.450	−.762	−.405	−.837	−.587	−.093	−.903	−.540	−.268	−.573	−.268	.908	.871	−.165	.424	1.000							
Bio17	−.288	.115	−.103	.343	−.215	−.493	.274	−.198	−.379	−.183	−.379	.258	.214	.973	−.862	−.112	1.000						
Bio18	−.397	−.661	−.256	−.880	−.547	−.059	−.879	−.501	−.209	−.537	−.209	.844	.895	−.069	.452	.940	−.057	1.000					
Bio19	−.288	.115	−.103	.343	−.215	−.493	.274	−.198	−.379	−.183	−.379	.258	.214	.973	−.862	−.112	1.000	−.057	1.000				
Alt	−.940	−.775	−.681	−.529	−.957	−.780	−.644	−.962	−.875	−.962	−.875	.469	.438	.159	.184	.542	.104	.508	.104	1.000			
Rddes	.139	.075	−.087	.285	.185	.074	.226	.164	.075	.178	.075	−.397	−.424	−.310	.119	−.296	−.326	−.378	−.326	−.153	1.000		
Rvdes	.351	.606	.332	.678	.442	.057	.697	.438	.211	.457	.211	−.331	−.237	.460	−.577	−.547	.470	−.470	.470	−.486	.200	1.000	
Setdes	.370	.230	.163	.236	.369	.311	.235	.377	.333	.379	.333	−.310	−.281	−.152	.002	−.295	−.168	−.272	−.168	−.378	.489	.206	1.000
